# Improved Genome Sequence of Wild Emmer Wheat Zavitan with the Aid of Optical Maps

**DOI:** 10.1534/g3.118.200902

**Published:** 2019-01-08

**Authors:** Tingting Zhu, Le Wang, Juan C. Rodriguez, Karin R. Deal, Raz Avni, Assaf Distelfeld, Patrick E. McGuire, Jan Dvorak, Ming-Cheng Luo

**Affiliations:** * Department of Plant Sciences, University of California, Davis CA 95616,; † School of Plant Sciences and Food Security, Tel Aviv University, Tel Aviv, Israel

**Keywords:** *Triticum dicoccoides*, Genome assembly, DLS, Pseudomolecules

## Abstract

Wild emmer (*Triticum turgidum* ssp. *dicoccoides*) is the progenitor of all modern cultivated tetraploid wheat. Its genome is large (> 10 Gb) and contains over 80% repeated sequences. The successful whole-genome-shotgun assembly of the wild emmer (accession Zavitan) genome sequence (WEW_v1.0) was an important milestone for wheat genomics. In an effort to improve this assembly, an optical map of accession Zavitan was constructed using Bionano Direct Label and Stain (DLS) technology. The map spanned 10.4 Gb. This map and another map produced earlier by us with the Bionano’s Nick Label Repair and Stain (NLRS) technology were used to improve the current wild emmer assembly. The WEW_v1.0 assembly consisted of 151,912 scaffolds. Of them, 3,102 could be confidently aligned on the optical maps. Forty-seven were chimeric. They were disjoined and new scaffolds were assembled with the aid of the optical maps. The total number of scaffolds was reduced from 151,912 to 149,252 and N50 increased from 6.96 Mb to 72.63 Mb. Of the 149,252 scaffolds, 485 scaffolds, which accounted for 97% of the total genome length, were aligned and oriented on genetic maps, and new WEW_v2.0 pseudomolecules were constructed. The new pseudomolecules included 333 scaffolds (68.51 Mb) which were originally unassigned, 226 scaffolds (554.84 Mb) were placed into new locations, and 332 scaffolds (394.83 Mb) were re-oriented. The improved wild emmer genome assembly is an important resource for understanding genomic modification that occurred by domestication.

Sequencing of wheat genomes has until recently been hampered by polyploidy, the large genome sizes, and high percentages of repetitive DNA. The first attempt to assemble the hexaploid wheat genome sequence using the whole-genome-shotgun (WGS) approach (Brenchley *et al.* 2012) met with only moderate success. Technological advances since then, such as improved mate-pair libraries, a new assembly algorithm implemented in the MAGIC assembler (NRGene, Nes Ziona, Israel), Hi-C technology (Lieberman-Aiden *et al.* 2009), and high-density genetic maps, have made it possible to produce a reference-quality WGS assembly, as demonstrated by WGS *de novo* assembly of the tetraploid genome of wild emmer wheat (Avni *et al.* 2017).

Wild emmer (*Triticum turgidum* ssp. *dicoccoides*, subgenomes BBAA) is the progenitor of all cultivated tetraploid wheat. Hexaploid bread wheat (*T. aestivum*, subgenomes BBAADD) evolved via hybridization of cultivated tetraploid wheat with *Aegilops tauschii* (genomes DD) (McFadden and Sears 1946; Dvorak *et al.* 2012). Wild emmer is therefore the wild ancestor of the A and B subgenomes of bread wheat.

The assembly of wild emmer accession Zavitan (WEW_v1.0) was by all measures a milestone that opened the door to the assembly of reference-quality genome sequences for other polyploid wheats. However, as is true for all first genome drafts, the WEW_v1.0 assembly has its limitations. The alignment of WEW_v1.0 pseudomolecules on a Bionano Genomic (BNG) optical map revealed the presence of incorrectly placed, incorrectly oriented, or chimeric scaffolds in the WEW_v1.0 assembly (Dvorak *et al.* 2018a,b).

The central feature of Bionano optical mapping technology is electro-kinetic aligning of labeled DNA molecules in nano-channel arrays for precision optical scanning. Our first attempt to produce a genome-wide optical map of the wild emmer genome (Dvorak *et al.* 2018a,b) employed DNA molecules nicked with a single-strand nicking restriction endonuclease followed by fluorescent labeling of the nicks (Das *et al.* 2010) and optical imaging of the labeled restriction sites (Lam *et al.* 2012). The BNG nick, label, repair, and stain (NLRS) chemistry has since been replaced by direct label and stain (DLS) chemistry. The DLS chemistry does not nick DNA, which eliminates the site-specific breaking of labeled molecules intrinsic to the NLRS chemistry (Deschamps *et al.* 2018). The net result of deploying DLS is longer optical contigs and greater genome coverage. Because the assembly of an optical map and assembly of the DNA sequence are independent of each other, the optical map can be used as an independent representation of a genome sequence for scaffold validation, super-scaffolding, and gap-closing during sequence assembly (Nagarajan *et al.* 2008; Luo *et al.* 2017).

Here, we report the construction of a Bionano optical map for wild emmer accession Zavitan based on the DLS chemistry. We then used this map and the NLRS map we constructed previously (Dvorak *et al.* 2018a) to re-assemble wild emmer pseudomolecules and produce an improved version of the wild emmer genome sequence assembly.

## Materials and Methods

### Plants

The wild emmer accession Zavitan was collected at the Zavitan nature reserve in Israel (Avni *et al.* 2014) and used for the genome sequence assembly of WEW_v1.0 (Avni *et al.* 2017).

### Optical map construction using the NLRS method

The construction of this Zavitan optical map has been reported in detail earlier (Dvorak *et al.* 2018a) and only essential facts will be repeated here. The map was constructed using DNA of accession Zavitan. The nicking endonuclease was Nt.*Bsp*QI (New England BioLabs, Ipswich, MA). The nicked DNA molecules were stained according to the instructions provided with the Bionano Prep DNA Labeling Kit (Bionano Genomics, San Diego, CA), as described in detail in Luo *et al.* (Luo *et al.* 2017). The labeled molecules were optically scanned using the Irys system. A consensus map was *de novo* assembled with the Assembler tool in the Bionano Solve v3.2 package using significance cutoffs of *P* < 1 × 10^−10^ to generate draft consensus maps, *P* < 1 × 10^−11^ for draft consensus map extension, and *P* < 1 × 10^−15^ for the final merging of the draft consensus maps.

### Optical map construction using the DLS method

High molecular weight (HMW) DNA was isolated as described previously (Dvorak *et al.* 2018a). HMW DNA was labeled with the DLE-1 enzyme (Bionano Genomics, San Diego, CA) and stained according to the instructions in the Bionano Prep Direct Label and Stain (DLS) Kit (Bionano Genomics, San Diego, CA). The labeled molecules were scanned with the Saphyr system. The consensus optical map was *de novo* assembled with the Assembler tool in the Bionano Solve v3.2 package using significance cutoffs of *P* < 1 × 10^−10^ to generate draft consensus maps, *P* < 1 × 10^−11^ for draft consensus map extension, and *P* < 1 × 10^−15^ for final merging of the draft consensus map while choosing the “nonhaplotype”, “noES”, and “noCut” options.

### Scaffolding

**Figure 1:**
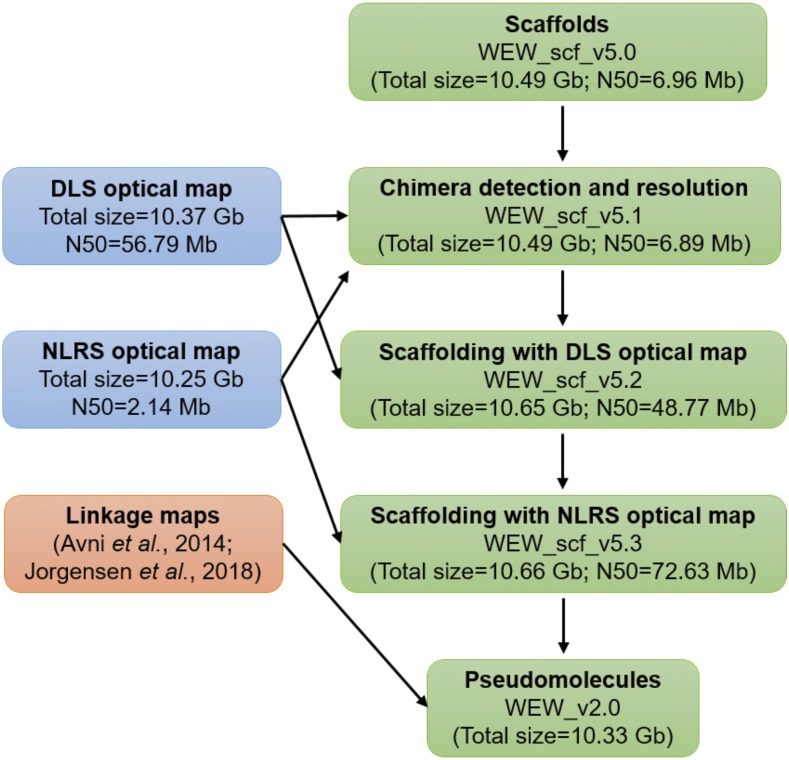
Overview of the strategy for the construction of WEW_v2.0 pseudomolecules. The major steps include scaffold chimera resolving, scaffolding with optical maps, and pseudomolecule construction.

The WEW_v1.0 sequence assembly (Avni *et al.* 2017) and wild emmer scaffolds (WEW_scf_v5) (Avni *et al.* 2017) were aligned on the DLS map using the RefAligner tool in the Bionano Solve package with an initial alignment cutoff of *P* < 1 × 10^−10^. If a conflict between the DLS map and sequence scaffolds was encountered, NLRS map was aligned to determine whether the inconsistency was due to an error in the sequence assembly or an error in the DLS map. Once all conflicts were resolved, scaffolding was performed using the Hybrid Scaffold pipeline in Bionano Solve v3.2 package (Bionano Genomics, San Diego, CA), with an alignment cutoff of *P* < 1 × 10^−10^. The gaps were filled with the number of Ns corresponding to the estimated length of a gap using flanking restriction sites. The workflow is illustrated in Figure 1.

### Pseudomolecule construction

The flow-sorted chromosome arm DNA (Chromosome Survey Sequencing, CSS) sequences (The International Wheat Genome Sequencing Consortium 2014) were used to assign scaffolds to the A and B subgenomes. High-density linkage maps of wild emmer and *Ae. tauschii* (Avni *et al.* 2014; Jorgensen *et al.* 2017; Luo *et al.* 2013) were used to determine the order and orientations of the scaffolds. The ordered and orientated scaffolds were then linked with 1000 Ns and anchored onto the 14 chromosomes (Figure 1**)**.

### Identification of homoeologous gene pairs between subgenomes

Sequences of 65,012 high-confidence (HC) genes annotated in the WEW_v1.0 assembly (Avni *et al.* 2017) were mapped to the new pseudomolecules using BLAT (Kent 2002) with default parameters. The top hits based on the identity and the coverage of each HC gene were retained. The gene set was then allocated to the A and B subgenomes and bidirectional BLAST (Altschul *et al.* 1990) was performed between the two groups with default parameters. The synteny analysis between the two subgenomes was performed using MCScanX (Wang *et al.* 2012) with default settings.

### Data availability

The genome assembly, including optical map, WEW_v2.0 pseudomolecules and unanchored scaffolds have been deposited under NCBI BioProject PRJNA310175. Supplemental material available at Figshare: https://doi.org/10.25387/g3.7459256.

## Results and Discussion

### NLRS and DLS optical maps

The map built previously with the NLRS system utilized 1,101 Gb of raw molecules (Table 1). The NLRS map consisted of 7,098 contigs with N50 = 2.14 Mb. The maximum contig length was 19.42 Mb. The total length of the map was 10.25 Gb, which is close to the total size of the sequence assembly (Avni *et al.* 2017).

**Table 1:** Characteristics of optical maps generated using different protocols

Feature	DLS protocol	NLRS protocol
**Enzyme**	DLE-1	Nt.*Bsp*Q1
**Molecule N50 (Kb)**	284	341
**Molecule minlen (Kb)**	150	180
**Molecule total length (Gb)**	1,107	1,101
**Coverage**	110x	110x
**# contigs**	601	7,098
**Max contig length (Mb)**	296.90	19.42
**Map total length (Gb)**	10.37	10.25
**Map N50 (Mb)**	56.79	2.14

The map built here with the DLS technology utilized a similar amount of sequence data (1,107 Gb), but in contrast to the NLRS map, it consisted of only 601 contigs with N50 = 56.79 Mb and total length of 10.37 Gb. The longest contig was 296.90 Mb.

Although the two maps were built from nearly identical amounts of data, the DLS map was far more contiguous than the NLRS map. In the NLRS method, DNA is labeled using a single-strand nicking endonuclease. When nicks are close to each other on the opposite strand of a double-strand DNA molecule, the nicking creates a fragile site, which is prone to a double-stranded DNA break. Such sites limit map contiguity. In contrast, the DLS chemistry labels DNA without nicking and does not produce systematic double-strand DNA breaks, and the contiguity of the DLS map is greatly improved. In our case, the N50 increased about 25-fold.

### Ambiguous regions in the WEW_v1.0 assembly

**Figure 2:**
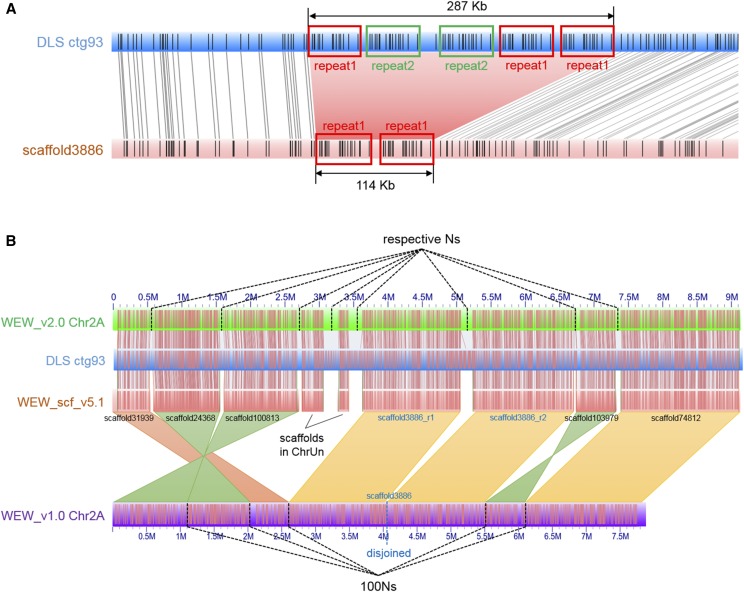
Detection of chimeras and reconstruction of pseudomolecules. (A) Discrepancy (pink shade) between scaffold3886 (pink rectangle) and DLS ctg93 (blue rectangle). Three copies of repeat 1 (red boxed) and two copies of repeat 2 (green boxed) were in tandem in an ∼287 kb region in DLS ctg93, but only two copies of repeat 1 were present in the ∼114 Kb region in scaffold3886, which was then disjoined into two scaffolds. (B) Illustration of pseudomolecule reconstruction. For a portion of the Chr2A (green rectangle) in the WEW_v2.0, 9 scaffolds of WEW_scf_v5.1 (pink rectangle) were ordered and oriented with the aid of DLS ctg93 (blue rectangle). In comparison, the portion in WEW_v2.0 is 9.1 Mb, whereas 7.9 Mb in the WEW_v1.0 (purple rectangle); three scaffolds (scaffold24368, scaffold100813, and scaffold103979) were re-oriented (green shades); three scaffolds (scaffold31939, scaffold24368, and scaffold100813) were re-ordered; scaffold3886 showed a discrepancy compared to DLS ctg93 (blue rectangle) and was disjoined (see detail in A); and two scaffolds of a total length of 490 kb in ChrUn of the WEW_v1.0 assembly were anchored onto Chr2A based on their alignments on DLS ctg93. The scaffolds in WEW_v1.0 were linked with 100 Ns, while they were linked with the number of Ns estimated with the optical maps in WEW_v2.0.

By aligning the 14 pseudomolecules of the WEW_v1.0 assembly onto the DLS and NLRS optical maps, numerous conflicting alignments were observed. Due to the limited ability of RefAligner software to align extremely long CMAPs (such as pseudomolecules in our case) with too many disagreements, all ambiguous sites could not be clearly seen and counted. Therefore, instead of the pseudomolecules, wild emmer scaffolds (WEW_scf_v5) from which the WEW_v1.0 pseudomolecules were built (Avni *et al.* 2017) were aligned on the DLS and NLRS optical maps. Among 3,102 scaffolds that could be aligned on the optical maps, only 47 scaffolds with 56 conflicts were found (Figure 2A), suggesting that the remaining conflicts in the WEW_v1.0 pseudomolecules were generated by incorrect ordering and orienting scaffolds during the pseudomolecule construction using the Hi-C method.

### Reconstruction of pseudomolecules

Since most errors in the WEW_v1.0 assembly were originated in the construction of the pseudomolecules, the pseudomolecules were reconstructed from the resulting scaffolds with optical maps. The 47 scaffolds with conflicting regions were corrected by breaking the sequences at positions containing Ns. Due to resolving these mis-assembled scaffolds, the number of scaffolds (WEW_scf_v5.1) increased from 151,912 to 151,968, and their N50 slightly decreased from 6,955,166 bp to 6,888,339 bp (Table 2). Hybrid scaffolding (see Methods) was first performed using the resolved scaffolds and DLS map. This produced scaffolds (WEW_scf_v5.2) with a total length of 10,650,512,398 bp and N50 = 48,768,823 bp (Table 2). By aligning the WEW_scf_v5.2 scaffolds to the NLRS map, they were validated and further scaffolded, which produced the assembly WEW_scf_v5.3. The WEW_scf_v5.3 contains 149,252 scaffolds with N50 of 72,632,893 bp and the longest scaffold being 278,440,484 bp (Table 2), approximately equivalent to the length of a chromosome arm.

**Table 2:** Scaffold characteristics at each step of their improvement with optical maps

Feature	WEW_scf_v5	WEW_scf_v5.1	Scaffolded using DLS map (WEW_scf_v5.2)	Further scaffolded using NLRS map (WEW_scf_v5.3)
**# sequences**	151,912	151,968	149,550	149,252
**Max length (bp)**	43,781,372	43,781,372	238,732,153	278,440,484
**Total size (bp)**	10,494,678,545	10,494,611,785	10,650,512,398	10,661,158,675
**Sequence N50 (bp)**	6,955,166	6,888,339	48,768,823	72,632,893
**N%**	1.63	1.63	3.07	3.30

The scaffolds in the WEW_scf_v5.3 were then ordered and oriented by using multiple high-density linkage maps (Avni *et al.* 2014; Jorgensen *et al.* 2017). A total of 485 scaffolds (10,330,081,199 bp) containing two or more SNP markers were anchored onto the 14 chromosomes (Table 3).

**Table 3:** Summary of the WEW_v2.0 and WEW_v1.0 pseudomolecules (Psm)

Psm	WEW_v2.0			WEW_v1.0		
	Length (bp)	Effective length (bp)	N%	Length (bp)	Effective length (bp)	N%
Chr1A	609,493,238	589,191,139	3.33	593,586,810	585,358,717	1.39
Chr2A	788,782,410	766,375,931	2.84	775,183,943	764,437,182	1.39
Chr3A	767,616,973	747,178,907	2.66	754,274,518	743,839,968	1.38
Chr4A	751,837,965	724,085,122	3.69	726,427,787	715,660,361	1.48
Chr5A	715,386,202	694,794,407	2.88	700,855,599	691,202,877	1.38
Chr6A	633,698,003	616,090,333	2.78	621,432,051	612,835,755	1.38
Chr7A	747,227,478	721,432,789	3.45	727,576,108	716,586,138	1.51
Chr1B	712,626,289	683,358,120	4.10	690,537,804	679,507,080	1.60
Chr2B	825,750,385	798,504,965	3.30	803,365,466	791,358,810	1.49
Chr3B	865,950,040	834,300,602	3.65	841,096,276	827,748,505	1.59
Chr4B	684,047,826	666,197,808	2.61	673,896,466	664,082,181	1.46
Chr5B	726,095,352	704,902,457	2.92	712,180,895	700,915,297	1.58
Chr6B	724,204,431	699,071,820	3.47	703,217,322	692,164,878	1.57
Chr7B	777,835,607	749,691,077	3.62	755,408,349	742,865,000	1.66
Total	10,330,552,199	9,995,175,477	3.25	10,079,039,394	9,928,562,749	1.49

### Assembly improvements

**Figure 3:**
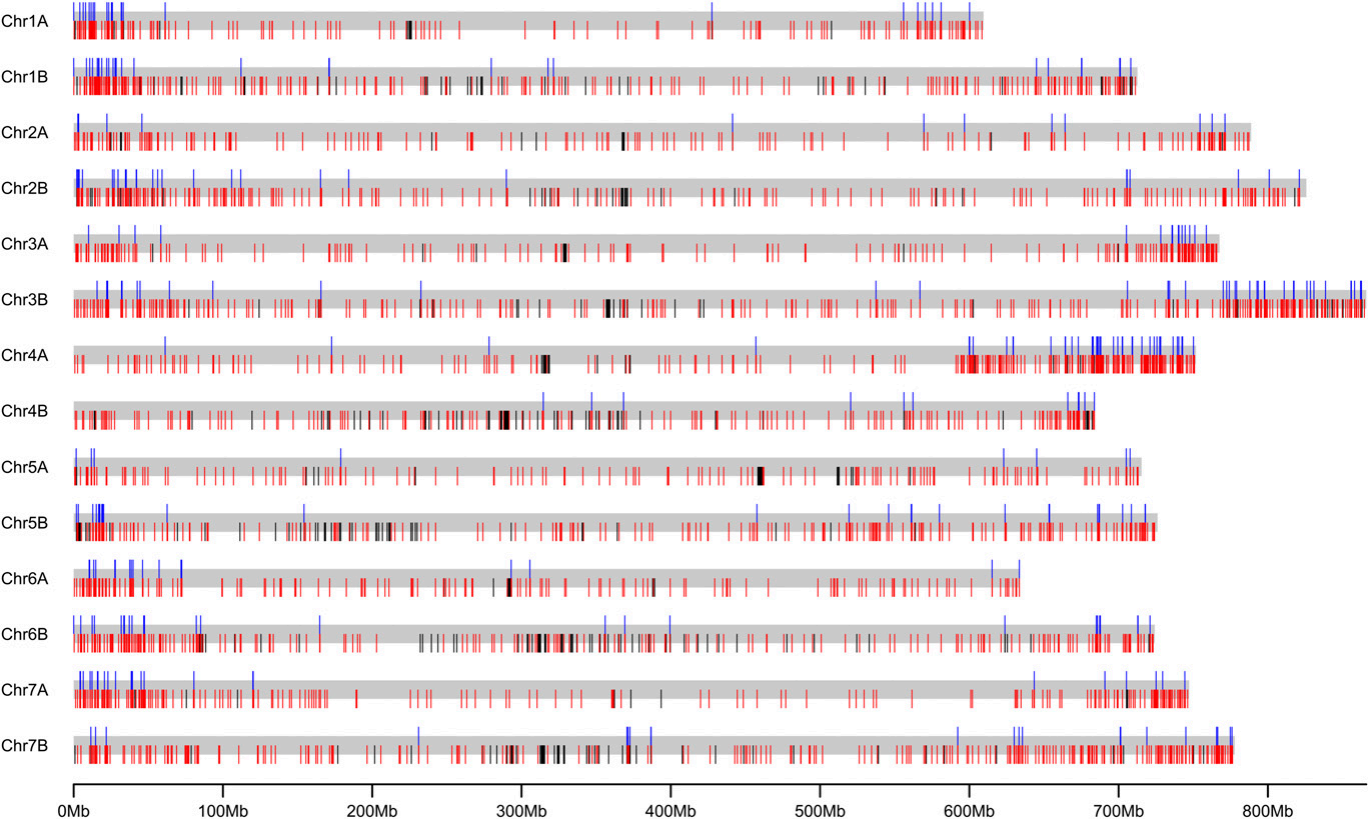
An overview of gap closing and gap size estimation in the 14 improved WEW_v2.0 pseudomolecules. Gray bars represent each of the 14 pseudomolecules. For each pseudomolecule, the upper ticks (blue) indicate ChrUn scaffolds of WEW_v1.0 assembly that were anchored onto chromosomes in the WEW_v2.0 pseudomolecules; the lower ticks in red indicate the gaps of unknown length in the WEW_v1.0 pseudomolecules that were estimated by optical maps in the WEW_v2.0 pseudomolecules; the lower ticks in black indicate gaps of unknown sizes in both versions of the pseudomolecules.

The WEW_v2.0 pseudomolecules are superior to the WEW_v1.0 pseudomolecules in the following ways. The effective lengths (excluding Ns) of the pseudomolecules were increased by approximately 67 Mb (0.7%), from 9,928,562,749 bp to 9,995,175,477 bp (Table 3), due to inserting 333 unanchored scaffolds into the WEW_v2.0 pseudomolecules (Table S1; Figure 2B; Figure 3).

There were 62,813 HC genes on the original WEW_v1.0 pseudomolecules. The remaining 2,179 HC genes were located on unassigned scaffolds (ChrUn) (Avni *et al.* 2017). In the WEW_v2.0 assembly, 64,992 HC genes were located on the pseudomolecules (Table 4), which represents an increase by 2,179 (3.4%) genes. For an unknown reason 20 genes that were originally annotated on WEW_v1.0 pseudomolecules could not be identified in the WEW_v2.0 pseudomolecules.

**Table 4:** Numbers of annotated high-confidence genes in each WEW_v2.0 and WEW_v1.0 pseudomolecule

Psm	WEW_v2.0	WEW_v1.0
**Chr1A**	3,974	3,804
**Chr1B**	4,441	4,232
**Chr2A**	5,121	4,963
**Chr2B**	5,834	5,544
**Chr3A**	4,731	4,565
**Chr3B**	5,254	5,072
**Chr4A**	4,523	4,350
**Chr4B**	3,725	3,639
**Chr5A**	4,900	4,818
**Chr5B**	5,168	5,026
**Chr6A**	3,685	3,594
**Chr6B**	4,315	4,187
**Chr7A**	4,817	4,636
**Chr7B**	4,504	4,383
**Total**	64,992	62,813

The number of gaps of unknown length was greatly reduced in the WEW_v2.0 pseudomolecules. There were 2,767 such gaps in the WEW_v1.0 pseudomolecules but only 471 in the WEW_v2.0 pseudomolecules (Table 5; Figure 3). The lengths of gaps between adjacent WEW_v1.0 scaffolds were unknown; they were uniformly filled with 100 Ns (Avni *et al.* 2017). In the WEW_v2.0 pseudomolecules, scaffolds were ordered and oriented prior to building the WEW_scf_v5.3 scaffolds using optical maps as guides. The alignments of WEW_scf_v5.3 scaffolds on the optical maps was employed in estimating the actual gap lengths between adjacent scaffolds (Figure 2B), which made the pseudomolecules a more realistic representation of the chromosomes. Reducing the number of gaps of unknown length also allowed a more accurate estimate of the size of the wild emmer genome, about 10.4 Gb.

**Table 5:** Numbers of gaps of unknown size in each WEW_v2.0 and WEW_v1.0 pseudomolecule (Psm)

Psm	WEW_v2.0	WEW_v1.0
**Chr1A**	15	133
**Chr2A**	15	157
**Chr3A**	15	167
**Chr4A**	26	209
**Chr5A**	21	141
**Chr6A**	11	144
**Chr7A**	10	172
**Chr1B**	48	233
**Chr2B**	34	218
**Chr3B**	44	270
**Chr4B**	57	209
**Chr5B**	48	218
**Chr6B**	60	235
**Chr7B**	67	261
**Total**	471	2,767

Ordering and orientating scaffolds with two independently constructed optical maps (Table S1; Figure 2B) is expected to reduce the rate of false positive discovery in studies of structural variation (Dvorak *et al.* 2018a). The WEW_v2.0 pseudomolecules had new locations of 226 scaffolds (554.84 Mb) that had been incorrectly placed, and re-oriented 332 scaffolds (394.83 Mb) that had been incorrectly oriented in the WEW_v1.0 pseudomolecules (Table S1). Errors in pseudomolecule assembly create false rearrangements which manifest themselves as shorter syntenic blocks containing fewer genes in comparisons of the A and B subgenomes. There were 45,141 HC genes in 7,230 syntenic blocks in the WEW_v1.0 pseudomolecules. In contrast, in the WEW_v2.0 pseudomolecules, there were 45,767 HC genes in 6,809 syntenic blocks, reflecting the improved scaffolds in WEW_v2.0.

In summary, we demonstrated the utility of optical maps for assembly of sequences of complex genomes. The DLS technology produced more contiguous maps than the NLRS technology. In turn, the deployment of DLS maps produce scaffolds with greatly improved N50. We should point out that the optical maps could not remove gaps within scaffolds that were inherited from the NRGene scaffolding in the WEW_v1.0 assembly. The replacement of those gaps by sequences should be the next objective of improving the Zavitan genome sequence assembly.

The wild emmer assembly WEW_v2.0 is now of comparable quality to assembly Aet_v4.0 of the genome of *Ae. tauschii*, the progenitor of the wheat D genome (Luo *et al.* 2017), produced with the aid of three different optical maps. The genomes of wild emmer and *Ae. tauschii* together represent the wild versions of the three subgenomes of the bread wheat genome, providing a reference for the bread wheat genome prior to its modification by domestication.
